# Innate Immune Cell Suppression and the Link With Secondary Lung Bacterial Pneumonia

**DOI:** 10.3389/fimmu.2018.02943

**Published:** 2018-12-14

**Authors:** David J. Morgan, Joshua Casulli, Christine Chew, Emma Connolly, Sylvia Lui, Oliver J. Brand, Rizwana Rahman, Christopher Jagger, Tracy Hussell

**Affiliations:** Manchester Collaborative Centre for Inflammation Research, The Lydia Becker Institute of Immunology and Inflammation, University of Manchester, Manchester, United Kingdom

**Keywords:** lung, macrophage, innate immunity, bacteria, virus, matrix, apoptotic cells, training

## Abstract

Secondary infections arise as a consequence of previous or concurrent conditions and occur in the community or in the hospital setting. The events allowing secondary infections to gain a foothold have been studied for many years and include poor nutrition, anxiety, mental health issues, underlying chronic diseases, resolution of acute inflammation, primary immune deficiencies, and immune suppression by infection or medication. Children, the elderly and the ill are particularly susceptible. This review is concerned with secondary bacterial infections of the lung that occur following viral infection. Using influenza virus infection as an example, with comparisons to rhinovirus and respiratory syncytial virus infection, we will update and review defective bacterial innate immunity and also highlight areas for potential new investigation. It is currently estimated that one in 16 National Health Service (NHS) hospital patients develop an infection, the most common being pneumonia, lower respiratory tract infections, urinary tract infections and infection of surgical sites. The continued drive to understand the mechanisms of why secondary infections arise is therefore of key importance.

## Introduction

It has been appreciated for a long time that infections following surgical cases are caused by a breach of skin barrier integrity. This breach of barrier tissue (e.g. the skin or epithelial surfaces lining the lung, gastro-intestinal or urogenital tract) however, is common during non-surgical infection and was one of the first causes identified to enhance bacterial outgrowth in the lung, by providing different substrates for adhesion and access to additional proteins for bacteria to metabolize. In this review we will discuss the changes in immunity that lead to dysregulation of responses and how prior viral infection in the lung suppresses cellular innate immunity facilitating bacterial outgrowth to occur. Though we assume that cellular innate immunity is adequate before viral infection, it is important to consider that patients most at risk of developing secondary bacterial complications may have a complex inflammatory history, medications, co-morbidities or mental-health history that has already influenced innate immunity. We will not cover the more soluble innate elements such as anti-microbial peptides or surfactant proteins, as these have been covered extensively elsewhere (1).

### Innate Immunity in the Healthy Lung

Innate immunity in the lung is important since it can facilitate the elimination of many pathogens in the absence of adaptive immunity and without immunopathological side effects. The actual location of some innate immune cell subsets is unclear due to the changing environment within the branching structure of the lung. A general rule however is that the density of immune cells gets lower the further down the respiratory tract you look, which facilitates optimal gaseous exchange.

As will be described for macrophages later, the immune components present in a healthy lung are specialized and sparse. Innate lymphoid cells exist in the naïve mouse lung at a low frequency of 0.4–1%. Their precise lung location in health however, has not been determined (2, 3), though they do expand during lung inflammation [for a review see (4)]. Gamma delta (γδ) T cells are also present and rare, accounting for ~1–5% of blood (5) and 8–10% of lung lymphocytes. They display a restricted profile of variable genes (V**γ**4, V**δ**1, and V**δ**6) (6) in their T cell receptor, which changes with age to become predominantly V**γ**4^+^ (7, 8). NK cells constitute 10% of resident lymphocytes in the lung (9) and it is thought their survival depends on IL-15 production by bronchial epithelial cells (10). NK cells detect an absence of MHC class I molecules using a variety of cell surface receptors and are induced to kill target cells by an activating receptor that binds stress ligands (11). In this way, NK cells present in the interstitial compartment are poised to recognize abnormality.

Dendritic cells (DCs) are present in the lung interstitial spaces (12) and the pulmonary epithelium (13), but are absent from the airspaces. In mice, DCs in the epithelium (CD103^+^ CD11b^lo^) require Batf3, IRF8, and Flt3 ligand for development, whereas those in the lung parenchyma require M-CSF (14). Either population may derive from bone marrow or a local precursor cell population (15). In the steady state, the DCs present in the epithelium may be important for sampling luminal content and/or clearing apoptotic cell turnover (16). As with other innate immune cells, the density of dendritic cells will depend on the position in the respiratory tree with more being present in bronchi than alveoli. Dendritic cells and follicular dendritic cells are also located in sparse B cell follicles. Though typically absent in naïve mice and humans, aggregates of B and T cells may be located next to the major bronchi and include follicular dendritic, dendritic, and stromal, cells (17).

### Macrophage Subsets in the Lung

Generally, an absence in any of the innate immune cells described above has little affect in healthy lungs. However, lung macrophages have a unique role in health by performing general housekeeping duties, as exemplified by the build-up of proteinaceous material due to an absence of macrophages in mice lacking granulocyte macrophage-colony-stimulating factor (GM-CSF) (18). In rodents and humans, the lungs are home to two distinct macrophage subsets: airway macrophages and interstitial macrophages (19). We refer to airway, rather than alveolar macrophages since bronchoalveolar lavage (BAL) samples the whole airway. This procedure typically elutes 90–95% macrophages in health, the majority of which will be derived from the alveoli, in addition to a small number of lymphocytes (20, 21). Alveolar macrophages are remarkably long-lived and self-renewing and therefore do not require continuous replenishment from bone marrow-derived precursors in health (22–24). In contrast, interstitial macrophages have a higher turnover rate and are shorter lived in the steady state (25). Interstitial macrophages are located in the interstitial space between the alveoli and capillaries and are less abundant than alveolar macrophages (26).

Alveolar macrophages are initially derived from fetal monocytes and their development is reliant on GM-CSF, of which there is an abundance of in the airspaces shortly after birth (27, 28). GM-CSF drives production of alveolar macrophages through induction of peroxisome proliferator-activated receptor-γ (PPARγ) expression (27, 29, 30). Mice lacking GM-CSF (or its receptor), and patients with defects in GM-CSF signaling, develop pulmonary alveolar proteinosis due to a build-up of surfactant in the airways because of a lack of clearance by macrophages (31, 32). Following irradiation (27) or influenza infection (22) airway macrophages become depleted and are replenished from the periphery or the interstitial lung macrophage pool, respectively. On the other hand, interstitial macrophages originate from bone marrow derived-monocytes and are preferentially replenished by this population during inflammation (33). A recent study has identified 3 populations of interstitial macrophages based on phenotypic and transcriptomic studies, which are different to airway macrophages (34).

#### The Function of Airway Macrophages

The mechanisms leading to bacterial outgrowth following lung viral infection are, to a large extent, driven by the attempt to return the lung to health. Understanding the role of innate immune cells in lung health therefore, may provide clues to why complications can occur. Due to their location, macrophages in the airways display phenotypic and functional differences to other macrophage populations. Alveolar macrophages reside in the alveolar lumen and are surrounded by surfactant, which contains proteins that dampen macrophage activity (35). This allows alveolar macrophages to be tolerant to cellular debris and innocuous antigens, thereby preventing excessive tissue damage, while setting an activation threshold that needs to be overcome to efficiently clear more pathogenic microorganisms (21). On the other hand, interstitial macrophages are in close contact with the extracellular matrix (ECM) and, as such, have a more prominent role in modulating tissue fibrosis, as well as being better equipped for antigen presentation (36, 37). Moreover, alveolar macrophages have reduced phagocytic activity and respiratory burst in comparison to interstitial macrophages (38, 39). Both subsets of macrophages inhibit T cell activation and subsequent onset of adaptive immunity via the suppression of DC activation; a process dependent on the anti-inflammatory cytokine interleukin-10 (IL-10), transforming growth factor-β (TGFβ) and prostaglandins (40, 41). Alveolar macrophages are poor at presenting antigen to T cells (42), although they are capable of transporting antigens to the lung-draining lymph nodes (43). Likewise, human alveolar macrophages induce T cell antigen-specific unresponsiveness as a result of poor antigen presentation and a lack of expression of co-stimulatory molecules, such as CD86 (44); which in itself promotes tolerance to innocuous antigens.

#### Regulation of Alveolar Macrophages by the Airway Epithelium

**Figure 1:**
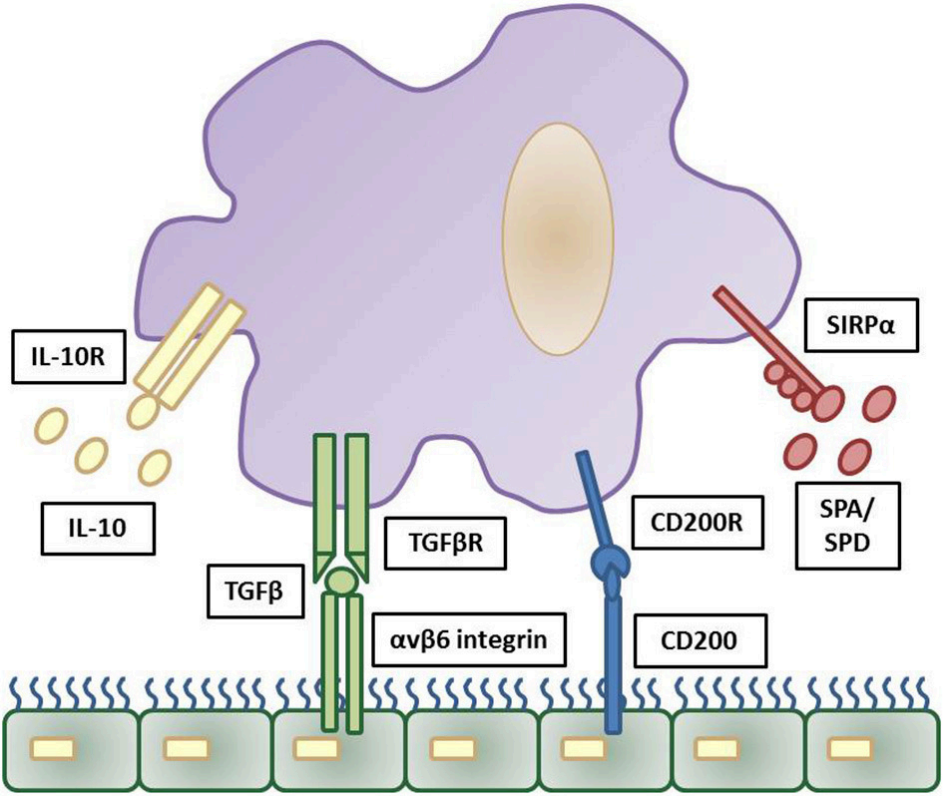
Inhibitory regulation of alveolar macrophages by the airway epithelium. Strict regulation of macrophage activation is required for homeostatic control of the general lung environment. As alveolar macrophages are under constant exposure to airborne endotoxins hypo-responsiveness is required for normal airway macrophage function. This is contributed through a number of downstream pathways triggered by airway epithelial cells production of IL-10, TGFβ, CD200, and surfactant proteins (SPA and SPD) and these reduce pro-inflammatory signaling and phagocytosis in airway macrophages via their respective cell surface receptors. The cascade of downstream inhibitory pathways to suppress macrophage activation are summarized elsewhere. Adapted from (35).

With respect to bacterial complications following viral infection, it is important to appreciate the role of the epithelium in regulating airway macrophage activity. Due to their direct exposure to environmental challenges in the alveolar lumen, strategies need to be in place for alveolar macrophages to discern a harmless antigen from a serious pathogenic threat. For this reason, alveolar macrophages are tightly regulated in order to prevent an inflammatory response against cellular debris and innocuous antigens, whilst still providing protection against harmful pathogens by propelling an inflammatory response (35). For example, alveolar macrophages are hypo-responsive to low levels of endotoxins, which are present in ambient air (21), thereby preventing an inappropriate innate immune response to innocuous antigens. A number of mechanisms are in place to suppress the activity of alveolar macrophages, including their interaction with the airway epithelium. The airway epithelium, through both direct contact and secreted products, negatively regulates alveolar macrophage activity. These factors include CD200, TGF-β, IL-10 and surfactant proteins (SP-A and SP-D), which act to suppress macrophage phagocytic ability and production of pro-inflammatory cytokines (45–47) (Figure 1). Though beneficial in some instances, these pathways can slow immediate immune activity. For example, knockout of IL-10 is beneficial as it allows immediate protection against acute influenza with better survival at lethal infection levels (48, 49). However, inhibiting IL-10 after acute influenza infection results in tissue inflammation and damage, with decreased survival (49, 50), similar to IL-10 knockout *S.pneumoniae* bacterial models (51). For an in-depth discussion on this see (48–51).

In addition, these mechanisms set a threshold of activation that needs to be overcome in order for an inflammatory response to be triggered. Activation of toll-like receptor (TLR) signaling, through recognition of an invading pathogen, elicits a strong enough immune response to exceed the inhibitory regulation of alveolar macrophages and causes up-regulation of TLR co-receptors including CD14 and triggering receptor expressed on myeloid cells 1 (TREM1) (52). Furthermore, loss of epithelial integrity during inflammation reduces the level of regulatory factors, releasing alveolar macrophages from epithelial-induced inhibition. This increases their phagocytic capabilities and initiates the production of pro-inflammatory cytokines (37, 53). The inhibitory factors that are important in maintaining airway homeostasis are also crucial in resolving inflammation after elimination of the microbial pathogen. Both CD200 and TGF-β assist in the suppression of inflammation, promote resolution and restore homeostasis (47).

### Dominant Viral Infections in the Lung

Human respiratory syncytial virus (hRSV), human rhinovirus (hRV), human parainfluenza virus (hPIV) and human metapneumovirus (hMPV), are the major types of viruses responsible for acute infections of the upper and lower respiratory tract (54). These respiratory viruses represent a significant burden on global public health, with acute respiratory tract infections (ARTIs) being the fourth highest cause of global mortality (55).

Influenza virus is a member of the orthomyxovirus family and a negative sense, single stranded RNA virus (56). The viral envelope of influenza virus is composed of haemagglutinin (HA) and neuraminidase (NA) (57), which are used as identifiers of virus subtypes (58, 59). There are four genera of influenza virus; A, B, C, and D, with the influenza A subtypes H1N1 and H3N2 causing the largest proportion of influenza cases (60). Influenza virus infection is one of the leading causes of respiratory tract infections worldwide, with ~5–20% of the global population infected and a mortality rate of up to 650,000 patients annually^1^ (61). The influenza virus predominantly invades human upper airway epithelial cells by binding to α-2,6 or α-2,3-linked sialy glycans expressed on their surface (62–64). The influenza virus can effectively evade detection by the host immune system. Genetic changes due to the error-prone nature of the viral RNA polymerase, that result in antigenic drift or recombination events between influenza viruses, can give rise to new subtypes of influenza that can lead to epidemic or pandemic outbreaks (65–67). Currently, our best options to combat influenza are by prevention using vaccines and treatment with antiviral medications. However, the variable nature of the virus limits the efficacy of both approaches as they need to be updated annually to keep up with the evolution of new subtypes (68).

hRSV is the main cause of acute lower respiratory tract infection (ALTRI) in infants, young children and older adults (aged ≥65 years) (69). hRSV is an enveloped negative-sense single-stranded RNA virus belonging to the Pneumoviridae family, Orthopneumovirus genus (69, 70). There are 2 major antigenic groups of hRSV, A and B, which can be further subdivided into 10 A genotypes and 13 B genotypes (71). The highly contagious nature of the virus means nearly all children will have been infected with hRSV by the age of 2 years old (72). Bronchiolitis or pneumonia caused by hRSV infection is the major cause of hospitalisations in children under the age of 2 years old. Additionally, hRSV infection has been implicated in the development of childhood asthma and recurrent wheezing (72–75). The global public health burden of hRSV is significant, with ~10% of all hospital admissions for severe bronchiolitis or pneumonia due to the virus, representing an annual cost of about 394 million USD (76–78). The severity of hRSV infection and associated clinical symptoms can be controlled by the use of palivizumab, a neutralizing monoclonal antibody to the fusion glycoprotein (F protein), which is a transmembrane surface protein in the viral envelope of hRSV (79–82). However, an effective vaccine against hRSV has yet to be developed.

The development of childhood asthma and recurrent wheezing is not only closely linked with infant hRSV-induced bronchiolitis, but is also associated with wheezing illnesses due to hRV infection in infancy (83–86). A member of the Picornaviridae, genus Enterovirus, hRV is a non-enveloped positive single-stranded virus (87, 88). hRVs can be classified into three species, with RV-A and RV-C, causing more severe respiratory illness, when compared to RV-B (88, 89). The species can be further categorized into genotypes, of which there are over 100 (87, 90). hRVs circulate throughout the year, are transmitted through direct contact or aerosol particles and are capable of infecting both the lower and upper respiratory tracts (87, 91, 92). Symptoms following infection are generally that of the common cold, including sore throat, cough, nasal congestion, sneezing and rhinorrhoea. However, in infants, the elderly, immunocompromised adults or those suffering from chronic respiratory illnesses, infection with hRV can be more severe. For example, hRV is responsible for 20–40% of all hospitalisations due to wheezing in infants aged 12 months or less (93, 94). Development of a vaccine and antivirals against hRV has been hindered by the vast quantity of genetically distinct genotypes (90, 95).

hPIV is second most common cause of ALTRI in children, after hRSV (96). hPIV, like hRSV, is an enveloped negative-sense single-stranded RNA virus of the Paramyxoviridae family (97–99). hPIV consists of four major serotypes—hPIV-1 and hPIV-3, genus Respirovirus and hPIV-2 and hPIV-4, genus Rubulavirus (100). By the age of 2 years old 60% of children have been infected by hPIV-3 and at the age of 5 years the majority have been infected by hPIV-1, hPIV-2 and hPIV-3 (97, 101). Although hPIV has been predominantly viewed as a cause of respiratory illness in pediatric patients, both immunocompromised and older adults are also susceptible to infection (97, 100). Clinical manifestations of infection by hPIV include the common cold, croup (laryngotracheobronchitis), tracheobronchitis, bronchiolitis and pneumonia (100). However, as of yet there is no effective antiviral treatment or vaccine available for hPIV.

Since its discovery in 2001, hMPV has been identified as one of the major causes of upper and lower respiratory tract infection in children, immunocompromised patients and the elderly, being detected in 4–16% of patients with ARTIs (102–108). hMPV, a negative-sense single stranded RNA virus, is a member of the Paramyxoviridae family, genus Metapneumovirus, and is closely related to hRSV and parainfluenza (108). Most infections with hMPV elicit mild to moderate clinical symptoms, although 5–10% of cases result in admission to pediatric intensive care (102, 107, 109).

### Bacterial Outgrowths in the Lung Following Viral Infection

A significant contributor to morbidity and mortality in respiratory viral infections is bacterial invasion. Given the colonization of the upper respiratory tract with common pathogens including *Streptococcus (S) pneumoniae, Haemophilus (H) influenzae* and most of the Staphylococcus species, a shift in immunological balance and the airway environment can undoubtedly cause severe secondary bacterial infection in the host. The most famous reports of bacterial colonization after lung viral infection stem from the 1918 influenza pandemic where between 20 and 60 million deaths were due to bacterial co-infection (110). It is estimated that ~25% of all influenza-related deaths are associated with co-infections, particularly during seasonal outbreaks (111, 112). Viral respiratory infections elevate nasopharyngeal bacterial density (113, 114), which may promote their colonization in the lower airways, though the precise mechanisms are unclear.

Bacterial co-infection is not limited to influenza virus. A retrospective cohort study of 6,000 hospitalized neonates in China showed that 94% had RSV infection, with the remainder having parainfluenza, influenza virus or adenovirus. The dominant co-infections in RSV infected neonates were *E. coli, Klebsiella (K) pneumoniae, S. aureus*, and *Enterobacter cloacae* (115). The high frequency of RSV and pneumococci co-infection in hospitalized children is reduced by prior pneumococcal conjugate vaccination and has led to the suggestion that treatment for secondary bacterial infections should be considered for pneumonia cases even if a child tests positive for RSV (116). The choice of antibacterial strategy may be critical since RSV can increase *S. pneumoniae* virulence by binding to penicillin binding protein 1a (117) and so penicillin derivatives may be ineffective. Experimental studies on human Rhinovirus 16 infection enhances *H. parainfluenzae, Neisseria subflava*, and to a lesser extent *S. aureus* in throat swabs (118). One study in adults revealed that rhinovirus was the most common (23.6%), then parainfluenza virus (20.8%), hMPV (18.1%), influenza (16.7%), and RSV (13.9%). However, virus strain occurrence may also be influenced by co-infections as RSV was significantly more common in those that also had community-associated pneumonia (119).

Bacterial and viral infections co-exist, and the post-viral bacterial outgrowths are often co-infections made up of different species of bacteria. In a recent meta-analysis, 28–35% of patients demonstrated positive laboratory culture with the co-infective species, *S. aureus* and *S. pneumoniae*, respectively (120). *S. pneumoniae* is the most common pathogen that causes community-acquired pneumonia and potential overwhelming sepsis, and is associated with high mortality and morbidity during influenza epidemics and pandemics (121, 122).

*S. aureus*, a gram-positive cocci and a common commensal in the nose and skin, is a major cause of bacteraemia (123). It is unclear why *S. aureus* has become a major cause of concern particularly in the pediatric population, of which a study of the 2003–4 season in the USA found that this organism not only dominated influenza-associated childhood mortalities, but was also found to be the most common causative bacterial agent in 46% of isolates, whereby more than 50% were methicillin-resistant strains (111). A rare and severe complication of community-acquired pneumonia is necrotising pneumonia, characterized by pulmonary consolidation, inflammation, necrosis, and ultimately gangrene, which is caused by methicillin-resistant *S. aureus*, a major public health concern due to its resistance to antimicrobials. Prior or co-infection with influenza infection and the presence of Panton-Valentine leukocidin (PVL) are both significantly associated with the necrotising pneumonia (124).

### Mechanisms of Bacterial Susceptibility After Lung Viral Infection

Other than a breach of the epithelial barrier, there are a number of modifications to cellular innate immunity in the lung that contribute to secondary bacterial infection.

#### The Role of Apoptotic Cell Clearance Following Viral Infection in Susceptibility to Secondary Bacterial Infections

**Figure 2:**
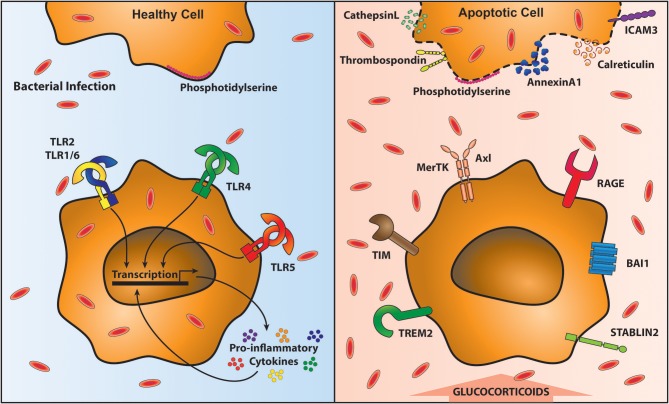
Clearance of apoptotic cells impairs anti-bacterial immunity. Removal of apoptotic cells requires their recognition by specialized receptors on phagocytic cells, including macrophages. In the presence of healthy cells (top left) Phosphatidylserine (PtdSer) is on the inner leaflet of the membrane. Local macrophages do not recognize them and therefore are able to signal through Toll-like receptors (TLR) unimpeded, resulting in the proinflammatory cytokine response. This optimal response is able to contain and clear bacterial infections (shown in red ovals). However, upon programmed cell death, PtdSer and a variety of other proteins are translocated to the outside of the cell membrane (top right). Macrophages recognize these exposed proteins via specific receptors (bottom right). These receptors facilitate apoptotic cell recognition and engulfment (known as efferocytosis) however, during efferocytosis macrophages are unable to respond to bacteria leading to their outgrowth (bottom right).

Cellular turnover by apoptosis features in health and inflammation. Airway macrophages play an important function in clearing apoptotic cells, a process known as efferocytosis, which is essential in maintaining airway homeostasis (125). Inefficient clearance of apoptotic cells leads to secondary necrosis and the release of damage associated molecular patterns (DAMPs) that subsequently promote an inflammatory response (126). Efferocytosis is mediated by a plethora of receptors that recognize externalized proteins on the cell surface of apoptotic cells. One of the most commonly studied proteins mediating efferocytosis is phosphatidylserine (PtdSer). PtdSer is present on the inner plasma membrane in living cells, but is externalized upon induction of apoptosis (127) by caspase inactivation of flippase (ATP11C) that is required to “flip” PtdSer back into the plasma membrane (128). Caspases also activate scramblases that “scramble” phospholipids in the plasma membrane; promoting exposure of PtdSer on apoptotic cells (129). Other proteins that flag up the presence of an apoptotic cell include oxidized low-density lipoprotein, calreticulin, annexin A1, ICAM-3, C1q, and thrombospondin (130). In parallel there are a number of receptors that recognize these proteins on apoptotic cells, including many that bind PtdSer: Triggering receptor expressed by myeloid cells-2 (TREM2) (131), CD300 (132), receptor for advanced glycation end products (RAGE) (133), Stabilin-2 (134), brain-specific angiogenesis inhibitor-1 (BAI1) (135) and TIM family members (T cell/transmembrane, immunoglobulin, and mucin) (136, 137) (Figure 2). For a review of other receptors recognizing externalized molecules on apoptotic cells see (130).

One PtdSer recognizing receptor family pertinent to the lung and its susceptibility to bacterial complications is the TAM receptor family (Tyro3, Axl, and Mertk receptors). These engulfment receptors require bridging molecules to link them to externalized PtdSer; protein S or growth arrest specific 6 (Gas6) (138). MerTK is ubiquitously expressed on macrophages, and even used as a defining marker for them. Axl, however, shows a more restricted distribution and is constitutively expressed on airway macrophages driven by GM-CSF and up-regulated during viral infection (139). Ligation of TAM receptors, in the presence of type 1 interferons (IFNs) enhances the expression of suppressor of cytokine signaling (SOCS) 1 and SOCS3, which reduce TLR and cytokine receptor signaling pathways (140–142). Furthermore, signaling via TAM receptors also induces TGFβ, IL-10 and prostaglandin production (143–146). This anti-inflammatory airway macrophage state is important to tolerate self-cells (125) but also reduces responses to subsequent coinfections (see Figure 2). Expression of IL-10 is raised following secondary bacterial coinfection after influenza virus exposure (147, 148). This is likely designed to prevent further tissue damage and to allow a return to homeostasis.

Lung viral infection enhances the apoptotic load due to cytopathology of infected cells and also the requirement to clear the large recruited immune cell infiltrate (149, 150). An absence of the TAM receptor Axl leads to excessive weight loss upon influenza infection in mice (139) that is likely linked to heightened secondary necrosis, which liberates DAMPS (151) recognized by pattern recognition receptors (PRRs), such as RAGE and ST2 (151–153). Axl knockout mice display increased nucleosome release in the airways corroborating the idea of enhanced secondary necrosis. This propagation of severe inflammation is likely to damage the lungs further and enhance the likelihood of secondary bacterial infections. Supporting this idea, prior exposure of mouse airway macrophages to apoptotic cells results in suppression of FcR-mediated phagocytosis and killing of bacteria. Furthermore, intrapulmonary administration of apoptotic cells impairs *S. pneumoniae* clearance from the infected lung (154). Also, suppression of antimicrobial responses of airway macrophages is enhanced by glucocorticoids, which promote efferocytosis, and treatment of mice with apoptotic cells in the presence of glucocorticoids is associated with elevated bacterial burden in the infected lungs (155).

Therefore, the normal process of clearing dying cells can have long term consequences and is particularly evident in chronic lung diseases (156) [for a review see: (157)]. However, further studies are required to determine the importance of this process, including analysis of the redundancy between apoptotic recognition receptors and the long term outcome of their manipulation. Efficient clearance of apoptotic cells may therefore provide an opportunity for therapeutic manipulation to lessen the severity of lung viral infections and prevent bacterial complications. In addition to the clearance of apoptotic cells by phagocytes, the phagocytes themselves (neutrophils and macrophages) may also undergo apoptosis.

#### Reduced Responsiveness of PRRs Following Viral Infection

Another natural process that occurs following viral infection is the cessation of inflammation. This is particularly important to allow efficient repair. Therefore, a prolonged inhibition of innate immunity is a common occurrence. However, a timely response to bacterial infection is critical to limit the pathogen load. Any delay in early immunity results in logarithmically higher bacterial loads that are difficult to clear and cause extensive bystander tissue damage. PRRs are important in this regard, but may be impaired by previous or concurrent inflammatory conditions. PRRs are widely expressed in the lungs on airway epithelial cells, alveolar macrophages and DCs and their ligation leads to the release of cytokines, chemokines, eicosanoids and type I IFNs into the airspaces (158, 159). The kinetics of this initial inflammatory wave limits early pathogen replication (159) by recruitment of monocytes, neutrophils and natural killer (NK) cells. NK cells target infected airway epithelial cells that have lost or reduced MHC class I expression (160), whereas monocytes and neutrophils aid alveolar macrophages in removing infected dead cells (161) and co-existing bacteria. Furthermore, type I IFNs stimulate the production of interferon-stimulated genes (ISGs), leading to cell-intrinsic and extrinsic antiviral activity (162). However, many studies have observed that subsequent stimulation via PRRs is defective following lung viral infection. This effect is not restricted to PRRs as defects in multiple processes employed by the mononuclear phagocyte system have been observed (69).

Following an acute viral infection, mouse airway macrophages display a similar phenotype to those in health (CD11c, CD11b, F4/80, and Siglec F). However, their responsiveness to TLR agonists is significantly dampened (163). We called this “innate imprinting” in 2004 (164), which is similar to the concept of “trained immunity” described by others in which monocytes acquire a tolerant phenotype after stimulation (165–167). This un-responsive state has recently been described as “immune paralysis” (168). In addition to influenza virus, human rhinovirus infection also predisposes to bacterial infection via degradation of IRAK-1 (interleukin 1 receptor associated kinase) leading to enhanced infection of respiratory epithelial cells by *H. influenza* (169). Defective TLR signaling would clearly lead to a reduction in many aspects of inflammation. With respect to subsequent bacterial infections, however, the most damaging consequences are the IFNγ induced impairment of macrophage phagocytosis (170, 171) and the reduction in neutrophil recruitment due to suppressed IL-8 production. In addition to reduced recruitment, neutrophil function also appears impaired following viral infection with reported reductions in myeloperoxidase, reactive oxygen species and the bactericidal properties of neutrophil extracellular traps (NETs) [for a review see (172)]. Reduced recruitment of neutrophils would also impact on airway macrophage NLRP3 inflammasome activation that is important for the production of IL-1β (173–176).

Reduced TLR signaling during viral infection may contribute to the impairment of the IL-17 response required for bacterial containment. Th17 cells produce IL-17 and IL-22 and are regulated by IL-23 (177, 178). These cytokines are crucial for lung epithelial production of neutrophil recruiting chemokines and anti-microbial peptides (179). Influenza virus induced type 1 IFNs reduce IL-17, IL-22, and IL-23 and impair the clearance of *S. aureus*; an outcome that can be rescued by adenoviral delivery of IL-23 (180). Type 1 IFNs also impair IL-17 production from γδ T cells (180).

The anti-inflammatory state that occurs following lung viral infection creates some confusion as patients and mice that succumb to secondary bacterial infection ultimately display enhanced inflammation (147, 181–183). However, a sluggish immune response will ultimately lead to enhanced inflammation due to an exponentially higher bacterial load.

#### The Impact of Viral Infection on Other Airway Innate Immune Cells

In addition to viral induced modification of airway macrophages, other innate immune cells are also affected. Type-2 innate lymphoid cells (ILCs) increase during influenza virus infection and secrete IL-13 (3), which although important for wound repair, are not useful during bacterial infection. A similar population of Lin^−^ CD127^+^ ST2^+^ CRTH2^+^ ILC2s have also been identified in human lung tissue and BAL and are known to produce IL-13. In mice, methicillin-resistant *Staphylococcus aureus* induces IL-13 up to 3 days after influenza virus infection and impairs viral clearance. Later infection of MRSA after influenza however, exacerbates bacterial replication due to inhibition of IL-13 and an upregulation of IFNγ (184). A detrimental impact of IL-13 is also evident following chlamydia (185) and tuberculosis (186). IL-13 also promotes *Mycoplasma pneumoniae* and non-typeable *H. influenza* adhesion in cultured bronchial epithelial cells by increasing MUC18 (187) and overcomes the enhanced bactericidal effects on epithelial cells of beta-2 agonists (188). Collectively, these studies suggest that ILC2s can be beneficial or harmful depending on their kinetics.

Viral infection induces the early recruitment of NK cells to the lungs where they promote anti-viral immune cells through the release of cytokines and limit viral replication by removing infected cells that have down-regulated MHC class I. If NK cells are depleted, adaptive immunity is not optimal, which could lead to prolonged viral infection (189, 190). NK cells also influence dendritic cells to support Th17 and Th1 cells that are important in anti-bacterial immunity (191) and NK cell production of IL-22 is protective against Klebsiella lung infection (192). However, NK cells appear early in the antiviral response to lung viral infection and so may not be present during secondary bacterial infection. Indeed influenza virus is reported to decrease NK cells, which reduces clearance of *S. aureus* in a process dependent on TNF-mediated enhancement of macrophage phagocytosis (193).

IL-10 is upregulated by viral infection and dampens the activation of invariant natural killer T (iNKT) cells by inhibiting the production of IL-12 by lung monocyte-derived dendritic cells, which contributes to *S. pneumoniae* outgrowth (194). IFNγ increases susceptibility to secondary bacterial infection by promoting inflammation and damage in the upper respiratory tract through both the ligand IFNγ and IFNγ receptor (245). Though IFNγ stimulates a pro-inflammatory phenotype in alveolar macrophages, it inhibits bacterial phagocytosis (49)

Neutrophils are critical components of anti-bacterial immunity. In addition to their reduced recruitment due to impaired chemokine production, influenza virus also inhibits their activity by inhibiting Th17 cell induction of anti-microbial peptides (195). Viral induction of Setdb2 (a protein lysine methyltransferase) also represses the expression of the CXCL1 gene that recruits neutrophils (196) and defective G-CSF production impairs neutrophil digestion and/or killing of phagocytized bacteria via myeloperoxidase (MPO) activity (197).

γδ T cells are also important in susceptibility to secondary bacterial infections. These rare T cells directly recognize pathogen-associated molecular patterns (PAMPs), express a range of cytokine receptors that modulate their function, mediate cell cytolysis via FAS and TRAIL and release anti-microbial peptides and cytotoxic molecules. They also produce IFN-γ, TNF-α, and IL-17. γδ T cell IL-17 production is impaired during influenza infection by type I IFNs causing susceptibility to *S. pneumoniae* infection (198). The role of γδ T cells in the extent of lung inflammation during viral infection depends, however, on whether other underlying conditions are present. For example, γδ T cell depletion in murine models of rhinovirus infection in asthmatic mice enhances airway hyper-reactivity (199).

Mucosal-associated invariant T (MAIT) cells (200, 201) are a recently studied population that are important in mucosal tissues for anti-bacterial immunity. They express cytotoxic markers such as CD107a and granzyme B via synergistic actions of IL-12 and IL-7 (202) and produce IFN-γ, TNF-α, and IL-17A (203). Their role in the lung is beginning to emerge. Lower numbers of peripheral blood CD161(+)Vα7.2(+) MAIT cells are associated with fatality in hospitalized patients with avian H7N9 influenza (204). However, it is not currently known whether defects in this population may predispose to bacteria following virus infections in the lung.

### Wound Repair and Bacterial Susceptibility in the Airways

In addition to reduced neutrophil chemoattractants, the post-viral lung may be skewed toward wound repair that will not be conducive for bacterial recognition and clearance. The molecules mediating wound repair are often immune suppressive. IL-10 is enhanced following influenza infection and promotes bacterial replication in the post-influenza virus infected lung (148) by inhibiting multiple facets of immunity; a process that may be driven by the upregulation of indoleamine 2,3-dioxygenase (IDO) (205). Furthermore, regulatory T cells and TGFβ are raised post-viral infection to dampen inflammation and facilitate processes of wound repair; for example by inducing the synthesis of collagen (206). However, TGFβ is also anti-inflammatory and is required to limit the activity of dendritic cells (168). A recent study by the Schulz-Cherry group showed that knockout of the β6 integrin prevents the activation of latent TGFβ leading to the presence of constitutively activated airway macrophages (207). Wound repair therefore represents a double edged sword where anti-inflammatory components limit inflammation and promote repair, but at the same time leave hosts susceptible to bacterial infection.

A few studies have described that epithelial cell proliferation and the expression of lung repair genes are reduced following respiratory viral infection (208, 209). This implies that barrier repair is delayed, which may prolong the access to alternative adhesion and nutrition sources for bacteria.

The importance of the repair process in the outcome of viral and bacterial infection of the respiratory tract is elegantly illustrated by the administration of amphiregulin, which decreases inflammation and lung damage to influenza virus (3) and prevents mortality to a secondary bacterial infection in the absence of any discernible influence on bacterial load (208).

### Matrix, Innate Immunity, and Bacterial Adhesion in the Lung

Extracellular matrix is a highly organized structure containing precise patterning of 43 different types of collagen, 200 glycoproteins and 40 proteoglycans (210). These components combine to form the interstitial matrix and the basement membrane. Alterations in both of these impacts on the cellular content of the lung and airways, and the adhesion, growth and location of bacterial species.

The basement membrane contributes to tissue architecture and is a highly organized structure made up of collagen IV, laminins, proteoglycans (decorin, biglycan, aggrecan and versican), heparan sulfate proteoglycans (perlecan and agrin), and nidogen (211). Some of these components can bind to other proteins that have immune modulatory properties. Decorin and biglycan for example, bind TGF-β1 (212) and so any alteration of their density or position will impact on lung inflammation and tissue repair. Similarly, fibrillar collagens type I and III of the interstitial matrix, in addition to binding other collagens and ECM components, also interact with inflammatory cell surface receptors particularly integrins. VLA-1, for example, is expressed on influenza-specific lung CD8+ T cells and binds α1β1 on interstitial matrix facilitating retention of memory CD8+ T cells in the lung (213). It is not hard to imagine that matrix re-modeling due to viral infection will have numerous consequences, such as the retention of a higher immune cell burden (214). Those retained immune cells, however, may not be optimal for subsequent bacterial infections and may even hinder the early migration of anti-bacterial immunity. For a recent review on immune cell:matrix interactions in the lung see (215).

The degradation of matrix can also liberate bioactive fragments now called matrikines which have immune modulatory properties. For example, the proteolytic processing by matrix metalloproteinases, MMP8 and MMP9, of interstitial collagens liberates the bioactive fragment, acetylated tripeptide Pro-Gly-Pro (acetyl-PGP) which promotes lung neutrophil recruitment (216, 217).

Accumulation of extracellular matrix components requires additional effort from interstitial and alveolar macrophages to clear them. This renders them hypo-responsive to subsequent bacteria. Recently we have found that excess hyaluronan induces adverse events in this way (218). Hyaluronan is a glycosaminoglycan that is abundant in the lung interstitial matrix. It is extruded from cells by hyaluronan synthases forming long cable-like polysaccharide structures. Degradation of hyaluronan is mediated by hyaluronidases. High- and -low molecular weight hyaluronan is reported to be anti-inflammatory and pro-inflammatory, respectively (219). Furthermore, hyaluronan can be sampled in sputum and by bronchoalveolar lavage, suggesting accumulation in the airways (220, 221). We have recently reported that hyauronan continues to accumulate in the lung and airway long after resolution of acute influenza virus infection in mice due to excess production via HA synthase 2. Furthermore, this excess hyaluronan is cross-linked with inter-α-inhibitor heavy chains due to elevated TNF-stimulated gene 6 expression. IαI is a proteoglycan containing two heavy chains of ~80 kDa, and a light chain (bikunin) of ~25 kDa that confers protease inhibitory properties (222, 223). Circulating IαI leaks into tissues during inflammation. Its synthesis has also been described in lung epithelia where it mediates repair after lung injury (224). In our study, administration of intranasal hyaluronidase completely restored lung function without any deleterious side effects (218).

There are other examples of matrix alterations contributing to the pathogenesis of lung viral infections (225). Influenza infection induces the recruitment of myeloid cells expressing membrane type I matrix metalloprotease (MT1-MMP/MMP-14) that is important in lung development and homeostasis (226). MT1-MMP inhibition rescues tissue damage and mortality in influenza-infected mice and combined with the anti-viral, oseltamivir, affords complete recovery. Furthermore, MT1-MMP inhibition also prevents outgrowth of *S. pneumoniae* following influenza infection (227). The modulation of extracellular matrix may depend on the viral strain. Analysis of RNA datasets from patients infected with pandemic associated influenza strains shows that H5N1 and H7N9 infection are enriched for genes involved with the extracellular matrix pathway (228). The importance of lung recovery and resilience is also demonstrated in mice lacking endophilin B2 that display improved mechanosensing and collagen and elastin ECM remodeling compared to wild-type mice (229). There are many other examples where matrix and associated components impact on lung immunity, which have been comprehensively reviewed elsewhere (230).

In addition to viruses directly promoting bacterial adherence (e.g., the neuraminidase in influenza virus exposes bacterial attachment sites by cleaving sialic acids, which are also metabolized by bacteria as a food source (231)), viral induced changes in extracellular matrix will change the lung microbiome. Dysbiosis of microbial commensalism can significantly impact on the overall health and progression of disease. Bacteria and bacterial products induce phenotypic and functional changes in immune pro-inflammatory gene expression, cellular adhesion and migration, and cell death (232). Binding to the ECM allows bacteria to adhere to, and colonize, host tissue. In addition, bacteria demonstrate affinity for different matrix substrates and changes in ECM components may increase host-pathogen accessibility and increase of bacterial virulence (233).

A number of microbes have elastase activity and/or express binding proteins for elastin that aid their pathogenicity (234). *S aureus* binds to elastin rich sites and expresses elastin binding proteins (EbpS) which bind to soluble, but not structurally intact chains of elastin (234). The expression of EbpS is also associated with greater bacterial cell growth, promoting cell proliferation and colonization (234, 235) and evasion of phagocytosis (234). In addition, elastin proteolytic products induce MMP activity and a number of bacteria express elastases (234, 235) further promoting elastin availability and consequently bacterial binding.

*S. aureus* encodes the fibronectin binding proteins (FnBPs), MSCRAMM (microbial surface component recognizing adhesive matrix molecule) that adhere to fibronectin and fibrinogen (236). Since components of fibronectin influence TLR4 receptor signaling, FnBPs may also promote immune regulation (237). Bacteria express collagen receptors and their binding appears to depend on collagen fiber tensile strength, conformation and structural dynamics. In an *in vitro* model, applying increasing high tensile forces to collagen peptides restricts receptor binding, suggesting that structurally normal collagen fibers decrease available sites for bacterial adhesion. Injured states, where collagen fibers are cleaved by high MMP activity, may increase susceptibility toward bacterial colonization with reduced structural strength and increased accessibility for more bacterial binding capacity (238).

Von Willebrand factor (vWF) is a large multimeric adhesion molecule and stimulates adhesion of bacteria. In bacteria such as *S aureus*, adherence to host can also be mediated via vWF and bacterial binding protein staphylococcal protein A (SPA). SPA binds to soluble and insoluble forms of vWF, promoting bacterial attachment and enhancing virulence in the absence of immune cell detection and clearance (239).

Glycosaminoglycan (GAG) interactions are ubiquitously used for cellular and extracellular signaling in all biological processes. Microbes utilize this universal process of the host for binding, and colonization of the host environment. Bacteria express GAG species and different binding domains across their entire surface. Studies blocking, removing or decreasing expression of these GAG binding domains decrease bacterial virulence (attachment, colonization and infection) in a number of bacterial strains (240). Bacterial communities have different affinities for GAG species (240). A large study manipulating GAG binding domains showed that the removal of heparin sulfate in *S. aureus* and *S. pneumoniae* decreases bacterial attachment to lung epithelial cells and fibroblasts and the inhibition of synthesis produced the same effect (240). The normal GAG interactions of the host are also used by microbes to prevent immune detection and clearance. Bacteria such as Streptococcus coat their surface with soluble high molecular weight hyaluronan, inhibiting detection and clearance by macrophages (241). Degradation of hyaluronan from the host tissues or bacteria into the low molecular weight protein stimulates phagocytosis, demonstrating bacterial colonization and infection can be influenced by the processing of GAGs from both the bacterium and host (241).

### Fast Inflammation Is Good

**Figure 3:**
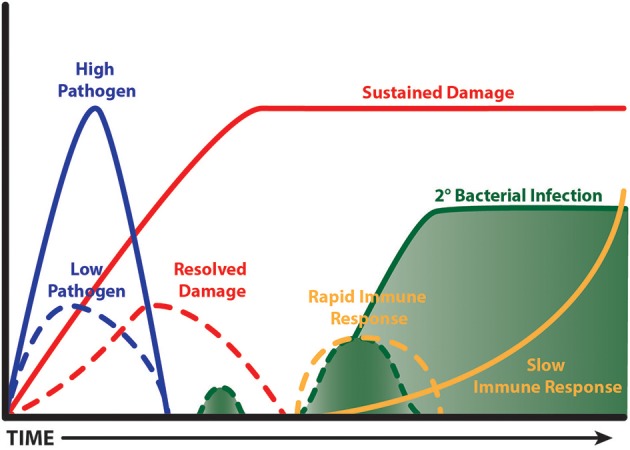
Fast and limited immunity is good. A time limited burst of inflammation limits bystander tissue damage, which in turn limits the extent of tissue repair. This leads to less impairment of anti-bacterial immunity and so a secondary bacterial infection is cleared. A virulent pathogen, or one that isn't cleared quickly, causes prolonged bystander tissue damage leading to a lengthy period of repair; the processes of which are anti-inflammatory. A subsequent bacterial infection is ignored and grows exponentially. Ultimately, innate immunity is activated when the bacterial load is excessive causing deleterious consequences.

Interestingly, a time limited burst of inflammation from the outset is beneficial during influenza infection in mice, which results in faster clearance and less collateral damage (Figure 3). The evidence to support this comes from detailed studies on IL-10 knockout mice and the response to pathogen clearance discussed earlier, and our studies with CD200 or CD200R knockout mice. CD200R signaling on myeloid cells limits inflammatory activity (242). Mice lacking CD200 or CD200R show heightened weight loss during influenza infection due to raised levels of inflammation (47). However, when these mice are next exposed to *S. pneumoniae*, they do not show susceptibility, because the first inflammatory event to influenza was quicker, thus causing less collateral damage (243). The benefit of a short burst of inflammation has recently been supported by data from the Metzger group where mice lacking SOCS-1 or IFNγ cleared influenza virus faster than littermate controls due to a rapid induction of immunity. By contrast, in the presence of SOCS-1, inflammation was prolonged and collateral damage increased (171). It would be interesting to test the impact of subsequent respiratory bacterial infection in the SOCS1 and IFNγ deficient model. Such studies might suggest that patients experiencing severe disease do so because their immune system is too sluggish. However, upon presentation at care facilities it would be too late to consider boosting immunity. The speed of immunity could possibly be specifically tackled in patients with other underlying conditions that render their innate immune system suppressed, as in the case of chronic obstructive pulmonary disease, or of the wrong phenotype to limit viral replication, as in the case of asthma. These patient groups are known to be at risk of severe viral infections [for example see (244, 245)].

The benefits of rapid induction of immunity to viral infection are also supported by research on IL-22. IL-22 is an interesting cytokine that is produced by innate immune cells and is critical for host protective immunity to lung *K. pneumoniae* (246), and *S. aureus* (183), but not to Mycobacterium tuberculosis or *M. avium* infection (164). IL-22 is upregulated during lung infection, but its neutralization has no effect on the kinetics of the disease or viral clearance. Rather it seems to function by promoting epithelial integrity and limiting lung damage (3, 247–249), which in turn prevents secondary lung infections by *S. pneumoniae* in mice (250). Interestingly, progesterone treatment of female mice also induces heightened IL-22 (and TGFβ and IL-6) and promotes faster recovery from influenza infection in female mice via epithelial production of amphiregulin. The resultant improvement of pulmonary function and reduced protein leakage is likely to diminish the risk of bacterial outgrowth, though this was not tested (251). In murine models of influenza infection, administration of GM-CSF promotes resistance to *S. pneumoniae* by promoting neutrophil recruitment and reactive oxygen species production from macrophages (252).

Another study that supports stimulation of immunity to prevent bacterial super-infections showed that the TLR-2 agonist, macrophage-activating lipopeptide 2 (MALP-2), reduces pneumococcal outgrowth in influenza virus infected mice (253). Also administration of nanoparticles containing the coat protein of a plant virus (papaya mosaic virus) and a single-stranded RNA causes the rapid recruitment of neutrophils, monocytes/macrophages and lymphocytes with beneficial effects on influenza virus and subsequent *S. pneumoniae* infection (254).

#### Creating a Debate in Matrix Modulation

Matrix modulation research is a field with great potential in restoring immune function via alternative key mechanisms. Extracellular matrix production is elevated following severe acute viral infection, which could have consequences on cell retention, immune paralysis of phagocytic cells and the physical properties of the airspaces into which it leaks. Respiratory fluids from COPD patients for example, contain higher levels of hyaluronan (HA) than healthy controls (59, 218) and we have recently shown this is exacerbated further by viral infection in COPD patients. Hyaluronidase treatment of mice after resolution of influenza virus infection restores lung function suggesting that the consequences of increased airway and lung hyaluronan is an impaired lung physiology (218). Airway hyper-reactivity is also improved during ozone-induced airway disease in CD44 or IαI deficient mice (60, 255) that bind HA or cross-link it, respectively. TNF-stimulated gene 6 catalyzes the transfer of IαI heavy chains onto HA (256) and TSG-6 null mice are resistant to airway hyporesponsiveness (257). Also TSG-6 promotes anti-inflammatory macrophages, (258) and inhibits neutrophil recruitment (259–262) and NFκB nuclear translocation. Just considering one matrix protein such as hyaluronan, the method of its production and degradation and the proteins that cross link it, provides multiple avenues for modulation. Therapeutic development in this area, to our knowledge, is poor with most focus on neutralizing enzymes that degrade matrix to prevent the liberation of small chemotactic matrix products. However, recombinant human hyaluronidase is licensed for therapeutic use in humans to increase barrier permeability, and although it is currently approved to enhance delivery and absorption of subcutaneous anesthetics, increase uptake of fluids, and to improve resorption of radiopaque agents (263, 264), it has the potential to be used to improve inflammatory diseases by immune-matrix modulation.

## Conclusion

Bacterial susceptibility following lung viral infection has been recognized for over a century and yet treatment options have not really altered since the introduction of antibiotics. It is now clear that long term suppression of innate immune mechanisms occurs following severe acute or chronic inflammation. In contrast to the clinical susceptibility toward bacterial infection that can occur in the 7 days following a viral infection, there are multiple long term modifications in immune mechanisms long after severe viral infections. These changes re-set the inflammatory tone of various immune cells by processes now known as trained immunity, innate imprinting or immune paralysis (164–166, 168). These molecular changes are evident during peak infection, but not in naïve un-infected lungs. This modified, tardy innate immunity immune response contributes to dysregulation of immune mechanisms to secondary bacterial exposure, rather than the clearance of the initial pathogen, and hence may explain the higher risk of long term bacterial outgrowth and chronic infection that cumulatively leads to excessive inflammatory disease. The majority of pathways leading to bacterial complications following viral infection have been discovered in single mouse strain studies. A recent report from the Metzger group shows that different mouse strains (BALB/C and C57BL/6) react differently to alveolar macrophage depletion following acute influenza infection. BALB/c mice respond to an acute influenza insult via IFNγ dependent alveolar macrophage depletion, whereas C57BL/6 mice do not. However, both are susceptible to post-viral bacterial coinfection (265). The precise combination of changes leading to bacterial super-infection may therefore be slightly different depending on genetic background.

Another area that requires development is that the known “at risk” patient groups currently identified for priority influenza vaccination (the elderly, asthmatic, pregnant etc.) do not account for the vast hospitalization numbers over the winter seasons. This suggests there may be other “at risk” groups.

This as exemplified by the rise of bacterial pneumonias in those experiencing low mood, stress, anxiety or mental health issues (266, 267). A mucosal barrier breach cannot explain all infectious complications. A population-based Danish study of 976,398 individuals, including 142,169 with a history of depression, onset of depression was associated with increased respiratory viral or bacterial complications (*IRR* = 1.58; *CI* = 1.36–1.85; *p* = 0.000) (268). Depression and stress are linked to suppression of multiple arms of innate and adaptive immunity [see (269) and references within], including a reduction of neutrophils (270) that are important for bacterial clearance. The link between mental health and infection is an area that will gain momentum in the next few years. Another area of concern that will likely garner research effort in the future is the influence of polypharmacy on respiratory infectious risk. In elderly patients hospitalized for pneumonia in Canada, 45% were taking 5 or more medications prior to hospital admission (271). A number of these medications may also modulate the immune system, though research in this area is sparse.

There is a window of opportunity between recovery from viral infection and the onset of bacterial outgrowth where innate immunity could be primed to react quicker. This may involve removal of immune suppressive pathways (CD200R, IL-10, and TGFβ), facilitation of apoptotic cell clearance (as apoptotic cell recognition receptors switch off innate immunity) or timely removal of high molecular weight matrix components from the airways. To identify these, studies are required that take into account other comorbidities, mental health status and the impact of polypharmacy on outcome.

## Author Contributions

EC, DM, CC, OB, SL, and TH drafted the manuscript. SL, OB, CJ, JC, and RR provided critical revisions and conceptual diagrams. All the authors made substantial contributions to the conception and design of the work, approved the submitted version of the manuscript, and agreed to be accountable for all aspects of the work.

### Conflict of Interest Statement

The authors declare that the research was conducted in the absence of any commercial or financial relationships that could be construed as a potential conflict of interest.

## Footnotes

^1^https://www.who.int/influenza/en/
